# Confounding factors in vesicle uptake studies using fluorescent lipophilic membrane dyes

**DOI:** 10.1080/20013078.2017.1388731

**Published:** 2017-10-12

**Authors:** Kaloyan Takov, Derek M. Yellon, Sean M. Davidson

**Affiliations:** The Hatter Cardiovascular Institute, University College London, London, UK

**Keywords:** Exosomes, extracellular vesicles, fluorescence, lipophilic dyes, CellMask, PKH67, lipoproteins, contaminants, serum

## Abstract

Small extracellular vesicles (sEVs) such as exosomes are nanocarriers of proteins, RNAs and DNAs. Isolation of pure sEV populations remains challenging, with reports of protein and lipoprotein contaminants in the isolates. Cellular uptake – a cornerstone for understanding exosome and sEV function – is frequently examined using lipophilic dyes such as PKH67 or CellMask to label the vesicles. In this study, we investigated whether contaminants can confound the outcomes from sEV and exosomes uptake experiments. sEVs were isolated from blood plasma of fasted or non-fasted rats as well as from serum-supplemented or serum-free conditioned cell culture medium using size-exclusion chromatography (SEC). Eluent fractions were characterized using nanoparticle tracking, protein and triglyceride assays and immunoassays. SEC fractions were labelled with different lipophilic dyes and cellular uptake was quantified using endothelial cells or primary cardiomyocytes. We report co-isolation of sEVs with apolipoprotein B-containing lipoproteins. Cellular dye transfer did not correspond to sEV content of the SEC fractions, but was severely affected by lipoprotein and protein content. Overnight fasting of rats decreased lipoprotein content and also decreased dye transfer, while late, sEV-poor/protein-rich fractions demonstrated even greater dye transfer. The potential for dye transfer to occur in the complete absence of sEVs was clearly shown by experiments using staining of sEV-depleted serum or pure protein sample. In conclusion, proteins and lipoproteins can make a substantial contribution to transfer of lipophilic dyes to recipient cells. Considering the likelihood of contamination of sEV and exosome isolates, lipophilic dye staining experiments should be carefully controlled, and conclusions interpreted with caution.

## Introduction

Interest in the biological functions of microvesicles and nanovesicles is growing exponentially in multiple scientific fields [1–7]. Different vesicle types are secreted from virtually all cell types to serve as extracellular mediators of short- and long-distance signals [8]. The smallest of them, and arguably the most studied, are exosomes. They are formed intracellularly within the multivesicular body and secreted by subsequent fusion of this organelle with the plasma membrane, thus releasing small 30–150 nm vesicles into the extracellular space [6,9–11]. Exosomes are found secreted in conditioned cell culture medium [12] and biological fluids such as blood [13], but a major pitfall remains the isolation of pure vesicle populations. Ample evidence exists for the presence of a considerable amount of contaminants in vesicle isolates obtained by all standard methods [14–17]. These include mainly non-vesicular proteins [14], RNAs [18–20] and lipoproteins [17,21–23] co-isolated with exosomes which may mask a true exosome-driven effect in functional studies *in vitro* or *in vivo*.

Another limitation lies in the mixed population of vesicles obtained in the isolates, prompting some authors to reconsider the term “exosomes” and instead refer to this vesicular population as “small extracellular vesicles (sEVs)” [24]. In this report, we will use the term sEVs for small vesicles isolated from blood plasma precleared of cells and large vesicles and isolated by size-exclusion chromatography (SEC).

Once released from cells, a potential fate of sEVs is cellular uptake and content delivery to recipient cells [25]. Intravesicular cargo including proteins, messenger RNAs, microRNAs, and single- and double-stranded DNAs [26–30] may induce profound alterations in the recipient cell physiology. Therefore, the visualization of vesicle internalization is considered to be a cornerstone for understanding sEV function. However, whether all cell types take up exosomes equally has not been established. Emerging evidence suggests that while endothelial cells are highly competent at exosome uptake, more differentiated cells such as cardiomyocytes may not be [31,32].

The most common approach to studying vesicle uptake involves labelling the sEV isolates with a fluorescent lipophilic membrane dye which should be incorporated into the membrane of sEVs. Some of the fluorescent dyes commonly used in the field of extracellular vesicle (EV) research include PKH26 [33], PKH67 [34,35], DiD [36,37] and CellMask [38]; however, all of them work by the same principle: they will readily be incorporated into any lipid structure, and none of them is specific to the vesicular membrane [39]. These dyes have been used widely in lipoprotein labelling [40–45] and can also bind to proteins [46]. Therefore, given the frequent co-isolation of lipoproteins and proteins with sEVs [21,47], it is important to assess the reliability of this method for tracking cellular sEV internalization.

SEC has been proposed as an effective means of purifying sEVs from plasma, at a purity comparable to density gradient purification [22,47–49]. We used sEVs obtained from blood plasma by SEC to study the potential contribution of non-sEV contaminants on fluorescent dye binding and cellular uptake. We showed that fluorescent transfer did not correlate with sEV content and observed similar or higher fluorescent uptake of various vesicle-poor fractions. Moreover, we directly stained serum and pure protein samples to demonstrate that both proteins and lipoproteins can make a marked contribution to lipophilic dye retention and transfer to acceptor cells. These findings are of importance for labelling experiments of plasma-derived sEVs and also of serum- or protein-supplemented cell culture media-derived vesicles.

## Materials and methods

### Animals and reagents

Male Sprague–Dawley rats (300–350 g) used in the experiments were obtained from Charles River, Margate, UK. Animals were treated in accordance with the Animals (Scientific Procedures) Act 1986 published by the UK Home Office. Food and water were provided *ad libitum*. Where indicated, a rat was fasted overnight (for approximately 18 h).

The reagents used for buffer preparation and cell collection and plating were all obtained from Sigma (Haverhill, UK) unless otherwise stated.

### Mouse cardiac endothelial cell (MCEC) line culture

To assess vesicular uptake into a cell line, an MCEC line derived from mouse neonatal microvascular cardiac endothelial cells [50] was purchased and cultured according to the manufacturer’s instructions (CELLutions, VH BIO LTD., Gateshead, UK). In brief, cells were maintained in Dulbecco’s modified Eagle’s medium (DMEM) supplemented with 25 mM glucose, 4 mM L-glutamine, 45 units/ml penicillin, 45 µg/ml streptomycin and 5% foetal bovine serum (FBS), and detached with 0.05% trypsin/0.53 mM ethylenediaminetetraacetic acid (EDTA) for passaging.

### Amniotic fluid stem cell (AFSC) culture

Spindle-shaped AFSCs were kindly provided by Dr Pascale Guillot Laboratory, University College London. AFSCs were maintained in DMEM supplemented with 25 mM glucose, 4 mM GlutaMAX (ThermoFisher), 45 units/ml penicillin, 45 µg/ml streptomycin and 10% FBS, and detached with TrypLE Express Enzyme (ThermoFisher, Dartford, UK) for passaging.

### Isolation of primary adult rat cardiomyocytes

To assess vesicular uptake into primary cells, we used adult rat ventricular cardiomyocytes isolated as described previously [31] with some modifications.

Buffer used for heart perfusion and cell collection (hereafter referred to as “Buffer”) contained 130 mM NaCl, 5.4 mM KCl (Fisher, Loughborough, UK), 1.4 mM MgCl_2_, 0.4 mM Na_2_HPO_4_, 4.2 mM 4-(2-hydroxyethyl)-1-piperazineethanesulphonic acid (HEPES), 10 mM glucose, 20 mM taurine (Acros, Thermo Fisher Scientific, Loughborough, UK) and 10 mM creatine. It was maintained at 37°C and pH 7.4.

Rats were anaesthetized with 250 mg/kg pentobarbital (Animalcare, York, UK) and 50 units heparin (Wockhardt, Wrexham, UK). Thoracotomy was performed and the heart eviscerated. After immediate cannulation of the aorta, the heart was retrogradely perfused with Buffer containing 750 µM CaCl_2_ (Fluka, Sigma Aldrich, Dorset, UK) followed by calcium-free Buffer. The heart was digested with Buffer containing 0.036% (w/w) collagenase (Worthington), 0.01% (w/w) protease and 100 µM CaCl_2_ (Digestion Buffer, Worthington, USA). After mechanical disruption and removal of the fibrous tissue, cardiomyocytes were collected with low-speed centrifugation and resuspended in M199 medium (Invitrogen, Waltham, MA, USA) supplemented with 5 mM creatine, 2 mM carnitine, 5 mM taurine (Acros), 50 units/ml penicillin and 50 µg/ml streptomycin. Cardiomyocytes were seeded on tissue culture plastic in defined areas preincubated with laminin.

### Blood plasma collection and preparation for SEC

Rats were anaesthetized with 250 mg/kg pentobarbital. Thoracotomy was performed and blood was collected from the inferior vena cava into a syringe filled with citrate buffer (final concentration ~15 mM). Blood was centrifuged at 1600 *g* for 15 min at room temperature to remove cellular material. The plasma was transferred to new tubes and centrifuged at 10,000 *g* for 30 min at room temperature to remove cell debris and large vesicles. Plasma was immediately processed for SEC fractionation.

### SEC of plasma and conditioned cell culture medium

Commercially available qEV SEC columns (Izon Science, Oxford, UK) were used to fractionate blood plasma or conditioned cell culture medium according to the manufacturer’s protocol [48].

For blood plasma SEC fractionation, 0.5 ml plasma aliquot was loaded on the qEV column (Izon Science) and the first 3 ml of eluent was discarded. Eluent fractions of 0.5 ml were then collected up to 17.0 ml and stored at −80°C.

For conditioned medium SEC fractionation, 4–6 × 10^6^ AFSCs were cultured for 24 h according to the aforementioned protocol either with 10% exosome-depleted FBS (ThermoFisher) or in serum-free conditions. Conditioned medium was collected and spun at 300 *g* for 10 min to remove dead cells. Supernatant was subsequently spun at 10,000 *g* for 40 min to remove cell debris and large vesicles. After discarding the pellet, medium was concentrated to approximately 300–400 µl using a Vivaspin 20, 100 kDa ultrafiltration unit (Sartorius, Epsom, UK). The remaining concentrate was loaded on a qEV column and the first 2.5 ml of eluent was discarded. Fractions of 0.5 ml were then collected up to 15.0 ml and stored at −80°C.

### Nanoparticle tracking analysis (NTA)

A NanoSight LM10-HS instrument (NanoSight, Malvern, UK) was used to measure the concentration and size of the particles present in SEC fractions and serum samples. Constant flow injection was used and three to five videos of 30 s were captured. The camera level was set at 15 and the detection threshold at 3–4.

### Transmission electron microscopy (TEM)

Electron microscopy was performed on a Jeol 1010 transmission electron microscope (Jeol, Warwickshire, Welwyn Garden, UK). Uranyl acetate (0.5%) was used to negatively stain the vesicles in the samples, as described previously [11].

### Protein assay

The bicinchoninic acid (BCA) assay was used to quantify the SEC fractions and serum protein content. First, 2–10 µl of each sample was added to a microplate and diluted to 10 µl (if necessary) with phosphate-buffered saline (PBS). Then, 190 µl of 49:1 bicinchoninic acid:copper sulphate solution was added to each sample and plates were incubated for 30 min at 37°C. Absorbance was read at 562 nm on a FLUOstar plate reader (BMG Labtech, Aylesbury, UK). Protein concentrations were calculated using bovine serum albumin (BSA) standards and a four-parameter logistic curve.

Conditioned cell culture media SEC fraction protein content was determined using a BCA protein assay kit for low concentrations (ab207002; Abcam, Cambridge, UK).

### Triglyceride assay

The triglyceride assay (Cayman Chemical, Ann Arbor, USA) was performed according to the manufacturer’s instructions. In brief, 10 µl of each sample was mixed with 150 µl of a triglyceride enzyme mix containing lipoprotein lipase, glycerol kinase, glycerol phosphate oxidase and peroxidase in 50 mM sodium phosphate buffer. The plate was incubated for 30 min at room temperature. Absorbance was read at 530 nm using a FLUOstar plate reader (BMG Labtech). Triglyceride concentration was calculated using manufacturer-provided standards and a four-parameter logistic curve.

### Dissociation-enhanced lanthanide fluorescence immunoassay (DELFIA)

Specific protein markers of sEVs and lipoproteins were quantified using a previously established DELFIA [22] with some modifications. In brief, 5–50 μl of each sample was diluted to 100 μl with PBS and added to high-binding microplates (R&D Systems, Abingdon, UK). The plate was incubated overnight at 4°C. After washing with DELFIA wash buffer (PerkinElmer, Beaconsfield, UK), blocking was performed with 100 μl 1% BSA/PBS solution for 1 h. Primary antibodies [CD9: Clone M-L13, BD Biosciences; CD81: Clone JS-81, BD Biosciences, San Jose, USA; HSP70: Clone N27F34, Abcam; and apolipoprotein B (APOB): Clone H-300, Santa Cruz Biotechnology, Santa Cruz, USA] were diluted in PBS and added at 1 μg/ml for 2 h. After washing, secondary goat anti-rabbit immunoglobulin G (IgG) (for APOB; catalogue number ab97073; Abcam) or goat anti-mouse IgG_1_ (for CD9, CD81 and HSP70; catalogue number ab98691; Abcam) conjugated to biotin was added to the plate and incubated for 1 h. Plates were washed and 1:1000 europium-labelled streptavidin in DELFIA Assay Buffer (PerkinElmer) was added and incubated for 1 h. After extensive washing, 100 µl DELFIA Enhancement Solution (PerkinElmer) was added to each well and the plate was shaken for 2 × 5 min at 300 rpm. Time-resolved fluorimetry was performed using a PHERAstar plate reader (BMG Labtech) with excitation at 337 nm, detection at 620 nm, integration start at 60 µs and integration time of 200 µs. Results are presented as arbitrary units (AU).

### Particle staining and cellular uptake

CellMask Orange fluorescent lipophilic membrane dye (ThermoFisher) was used to study the uptake of SEC fraction constituents in cell lines and primary cells. First, 50 µl aliquots of each indicated fraction from the SEC columns were stained with 7.5 µg/ml CellMask dye diluted in PBS in a final volume of 500 µl. The samples were incubated for 10 min at 37°C and transferred to 0.5 ml ultrafiltration units (Amicon, Merck, Kenilworth, UK; or Vivaspin, Sartorius) with a 100 kDa membrane. Three centrifugations of 5 min at 14,000 *g* were performed with the addition of PBS for each to wash the sample thoroughly and remove unbound dye. Concentrates were collected and about 15% of each was added per 2 ml of culture media to MCECs or primary adult rat cardiomyocytes. After incubation for 3 h in a conventional tissue culture incubator (37°C, 5% CO_2_), a Hoechst 33342 (ThermoFisher) was used to label the nuclei at 5 µg/ml. Five images of living cells in each group were then acquired on a Leica TCS SP5 confocal microscope using 40× (MCECs) and 63× (cardiomyocytes) magnification objectives and 543 nm (20%) and 405 nm (9%) lasers, with an Airy value of 1. Washing and fixation were performed with several additional samples which produced similar fluorescence signals (not shown). ImageJ software was used to measure uptake as whole image fluorescence intensity for MCECs or membrane fluorescence intensity for cardiomyocytes. Results are presented in AU. Independent experiments were also performed using different animal and isolation samples to ensure reproducibility (data not shown).

The same protocol was used for staining of serum and protein-only samples except that the starting aliquots were 20 µl. Commercial, exosome-depleted FBS was obtained from ThermoFisher. Exosome-depleted FBS, made in house, was prepared by overnight (18 h) ultracentrifugation (100,000 *g*, 4°C). Serum separation in FBS pellet and FBS supernatant was performed by ultracentrifugation of 1 ml of complete FBS at 100,000 *g* and 4°C for 70 min (plus an additional wash of the pellet under the same conditions).

For PKH67 staining, 100 µl of each indicated SEC fraction of serum-supplemented conditioned cell culture medium (exosome-depleted FBS; ThermoFisher) was stained with 3 µM PKH67 dye in Diluent C (Sigma) in a final volume of 500 µl for 10 min at room temperature. The samples were then processed as with the CellMask dye. Confocal imaging was performed using 488 nm (20%) and 405 nm (9%) lasers.

For DiD staining, 100 µl of each indicated SEC fraction of serum-supplemented conditioned cell culture medium (exosome-depleted FBS; ThermoFisher) was stained with 5 µM Vybrant DiD dye (ThermoFisher) in a final volume of 500 µl for 10 min at 37°C. The samples were then processed as with the CellMask dye. Confocal imaging was performed using 633 nm (20%) and 405 nm (9%) lasers.

## Results

### Particle and protein content of plasma SEC fractions

**Figure 1.**
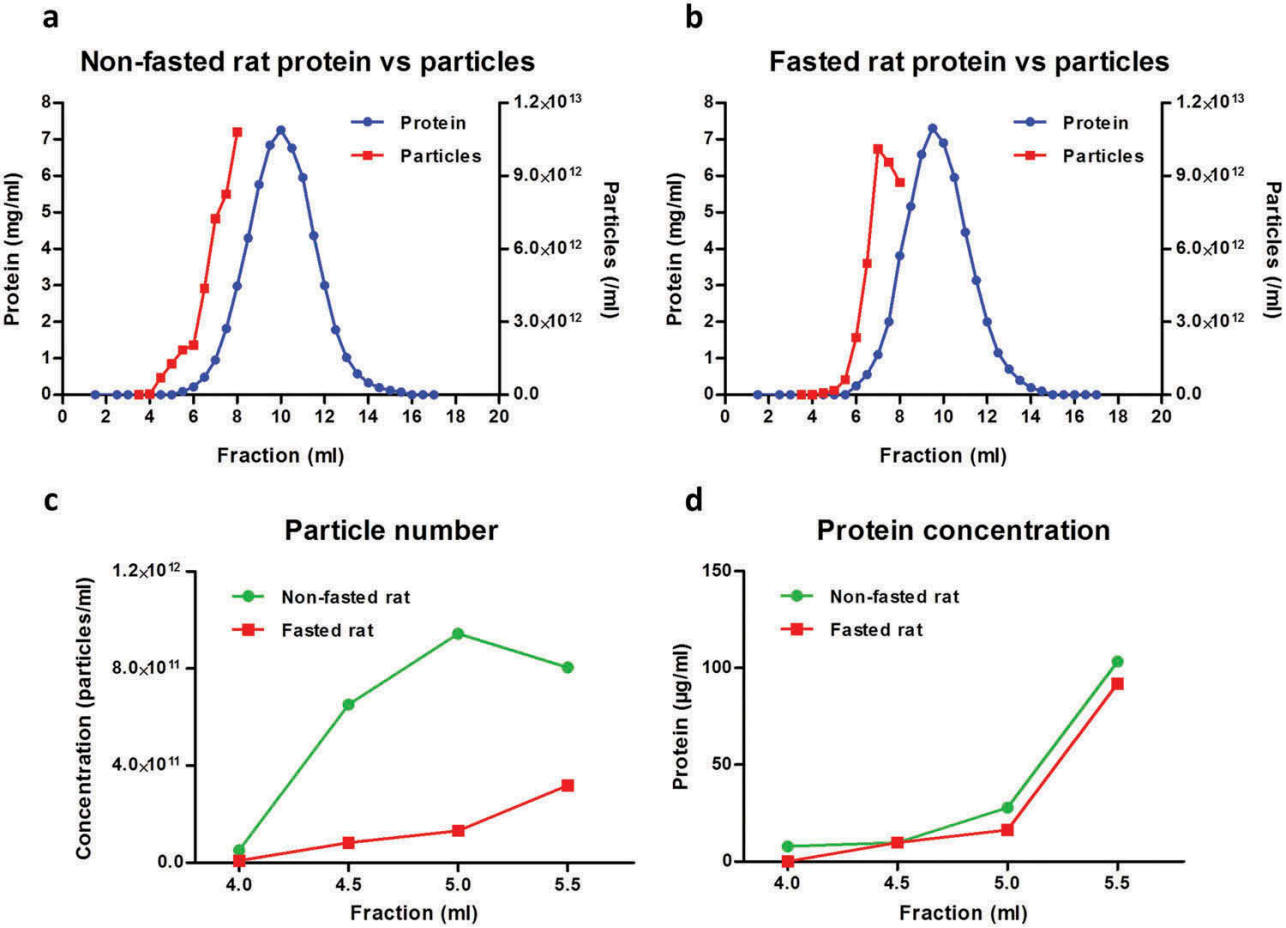
Particle and protein concentration in fractions obtained with size-exclusion chromatography (SEC) of rat blood plasma. Protein and particle concentration of 0.5 ml SEC fractions of blood plasma collected from (A) non-fasted or (B) overnight-fasted rats. First, 500 µl plasma precleared of cells, debris and larger vesicles was loaded on a qEV SEC column; 0.5 ml fractions were collected and protein content was measured. Nanoparticle tracking analysis was performed on fractions up to 8.0 ml using an LM10-HS NanoSight instrument. (C) Detail of particle concentrations in early fractions (4.0–5.5 ml) from (A) and (B). (D) Detail of protein concentration in early fractions (4.0–5.5 ml) from (A) and (B).

To investigate cellular uptake of fluorescently labelled plasma sEVs, it was necessary to obtain a representative population of sEVs purified using a standard, commonly used technique. For this reason, sEVs were isolated from rat blood plasma by SEC fractionation using commercially available SEC columns. In our hands, the majority of plasma proteins eluted between 7.0 and 13.0 ml, with a peak at 10.0 ml (Figure 1(A)). NTA showed that some particles eluted in the early, protein-poor fractions (< 6.0 ml), but a high concentration of particles was also detected in later, protein-rich fractions (Figure 1(A)). A possible limitation of SEC for isolation of sEVs from blood is the co-elution of large lipoproteins [e.g. chylomicron remnants, very low-density lipoprotein (VLDL), intermediate-density lipoprotein (IDL)] [22]. To reduce the amounts of these particles, rats were fasted overnight, as has been previously suggested [21]. This had a minor effect on the profile of protein elution of plasma (Figure 1(A,B)), but reduced the particle concentration in early fractions up to 5.5 ml (Figure 1(C,D); Supplementary Figure 1).

### sEVs co-isolate with APOB^+^ lipoproteins

**Figure 2.**
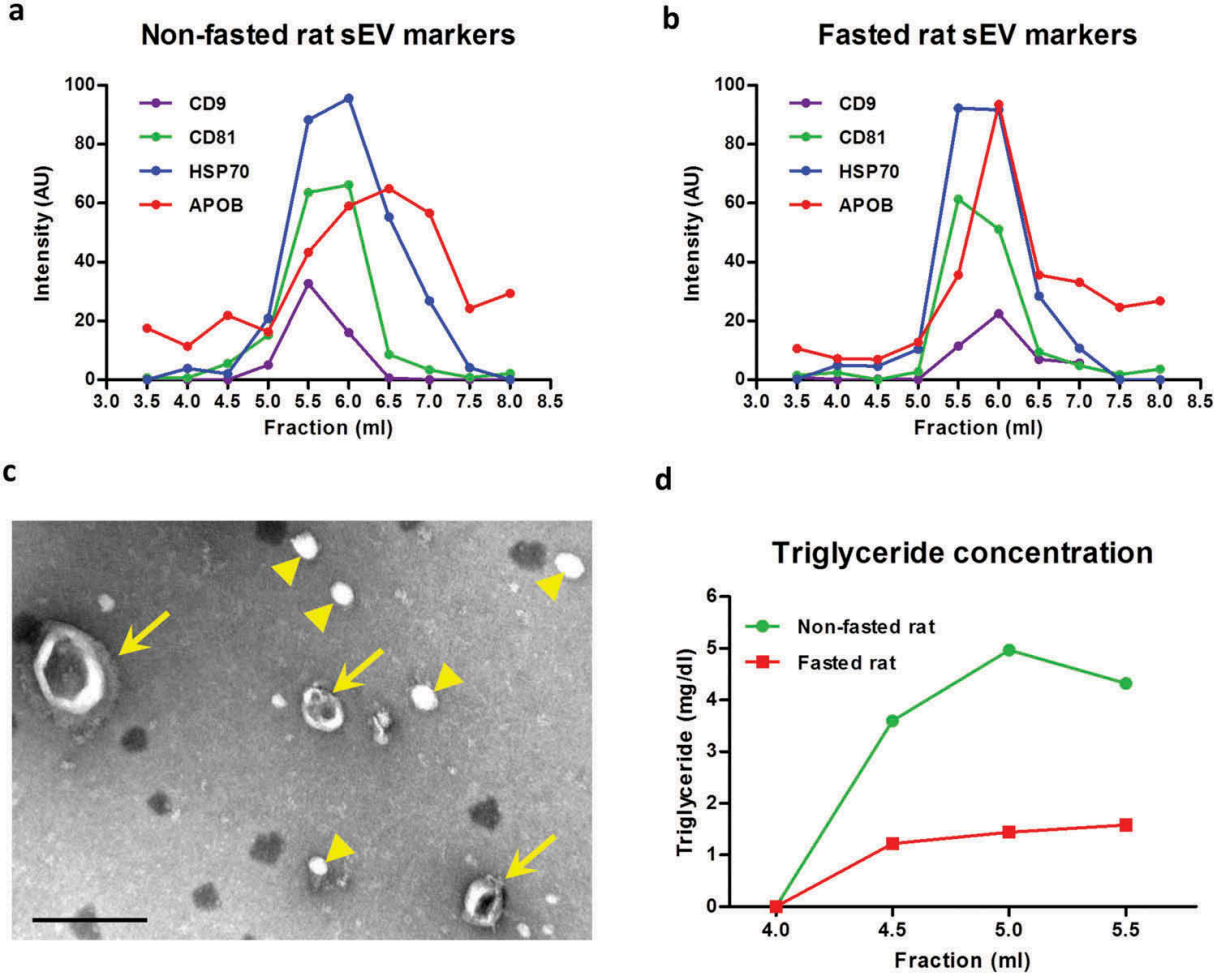
Small extracellular vesicle (sEV) and lipoprotein content in fractions obtained with size-exclusion chromatography (SEC) of rat blood plasma. Protein markers of sEVs (CD9, CD81, HSP70) and lipoproteins [apolipoprotein B (APOB)] were measured using a modified dissociation-enhanced lanthanide fluorescence immunoassay in SEC fractions 3.5–8.0 ml obtained from blood plasma of (A) non-fasted or (B) fasted rats [22]. AU, arbitrary units measured by time-resolved fluorescence. Assays were performed together and AUs from panels (A) and (B) can be compared. (C) Transmission electron microscopy image showing the presence of sEVs (arrows) and lipoproteins (arrowheads) in SEC fraction 5.5 ml collected from plasma of a non-fasted rat. Scale: 200 nm. (D) Triglyceride concentration of early fractions (up to 5.5 ml) collected from plasma of non-fasted and fasted rats.

To investigate the profile of sEV and lipoprotein elution in SEC fractions, an immunoaffinity DELFIA method was performed to measure sEV- or lipoprotein-specific markers. Vesicles bearing sEV marker proteins CD9 and CD81 eluted predominantly in fractions 5.5 ml and 6.0 ml for both fasted and non-fasted animals (Figure 2(A,B)). HSP70 protein, which is also localized on the sEV membrane [31], had an analogous peak elution (Figure 2(A,B)). APOB^+^ lipoproteins [i.e. chylomicrons, VLDL, IDL and low-density lipoprotein (LDL)] [51] co-eluted with the sEVs, although the peak was slightly delayed to 6.0–7.0 ml. Some APOB^+^ lipoproteins, possibly smaller LDL particles, continued to elute later at 7.5–8.0 ml (Figure 2(A,B)). TEM confirmed the presence of cup-shaped exosome-like vesicles in the sEV-rich fraction 5.5 ml, although abundant spherical particles resembling the TEM appearance of lipoproteins were also present (Figure 2(C)).

Triglycerides, here used as an overall indicator of lipoprotein content, were notably lower in fasted rat plasma (17.4 mg/dl) than in non-fasted plasma (56.3 mg/dl). Early SEC fractions from the fasted plasma also had lower triglyceride levels (Figure 2(D)) and fewer large lipoprotein-like particles visible on TEM (Supplementary Figure 2(A)). Late fraction 8.0 ml was enriched with protein and some small lipoprotein-like particles were evident in the TEM images (Supplementary Figure 2(B)).

In summary, we obtained good separation of CD9^+^, CD81^+^ and HSP70^+^ sEVs (in fractions 5.5–6.0 ml) from bulk protein (7.0–13.0 ml), but also observed co-elution of some APOB^+^ lipoproteins with sEVs.

### Endothelial uptake of lipophilic dye-labelled plasma-derived sEVs may be masked by the presence of contaminants

**Figure 3.**
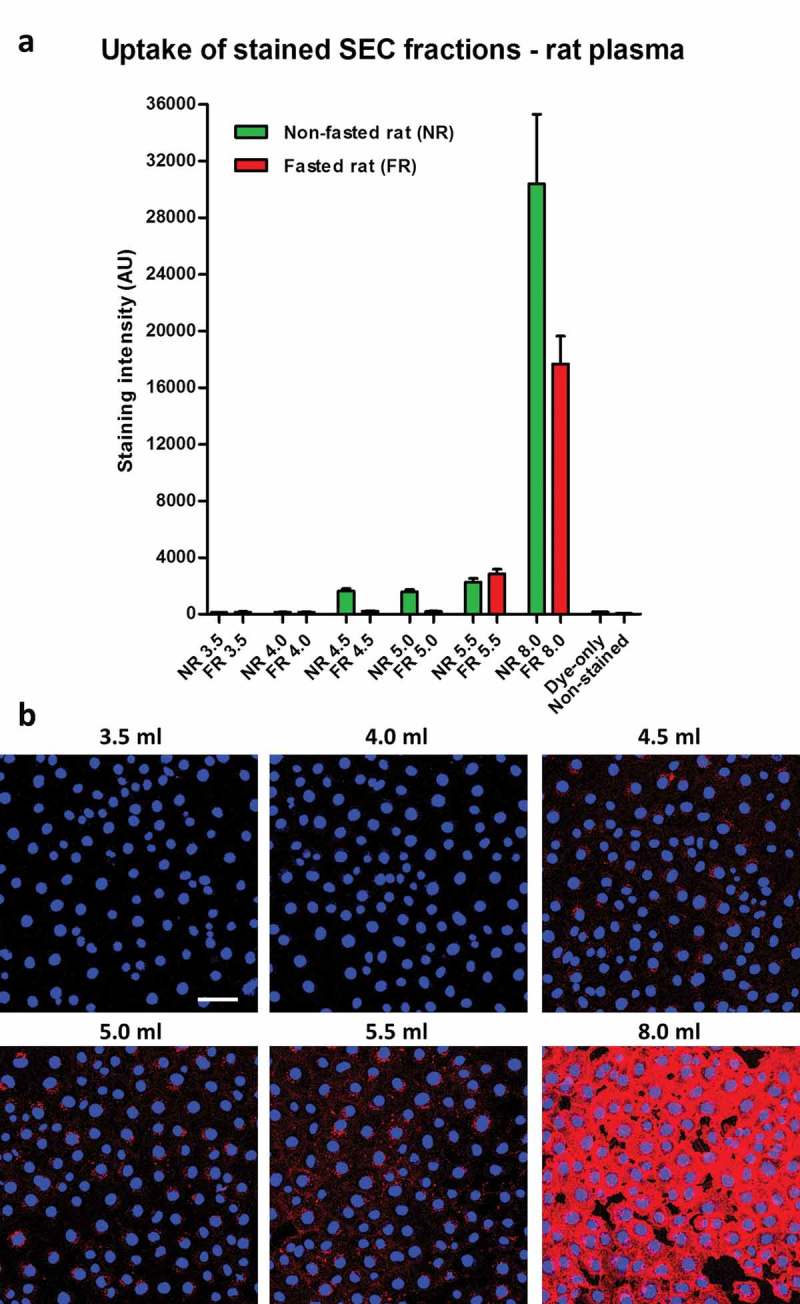
Uptake of lipophilic dye-labelled plasma size-exclusion chromatography (SEC) fractions into mouse cardiac endothelial cells (MCECs). (A) Equal volumes (50 µl) of SEC fractions 3.5–5.5 ml and 8.0 ml obtained from fasted and non-fasted rat blood were labelled with 7.5 µg/ml CellMask Orange lipophilic membrane dye and washed. Small aliquots of the stained material (~15%) were added to a confluent layer of MCECs and incubated for 3 h. The graphs show means with SEM of fluorescence intensity of five images obtained in a single experiment. NR, non-fasted rat; FR, fasted rat; followed by the respective SEC fraction in millilitres; Dye-only, phosphate-buffered saline negative control stained with the lipophilic dye; non-stained, background MCEC fluorescence without addition of a sample; AU, arbitrary fluorescence intensity units. (B) Representative images of fluorescence uptake of SEC fractions from plasma collected from a non-fasted rat. Scale: 50 µm.

A routine method for investigating internalization of sEVs includes incubation of cells with vesicles labelled with fluorescent lipophilic dyes. To investigate dye retention and transfer of vesicle-poor and vesicle-rich SEC fractions, the fractions were labelled with CellMask dye, and then extensively washed. Dye-labelled samples were then added to MCECs (a mouse cardiac endothelial cell line) and incubated for 3 h. Cellular fluorescent uptake was observed with the stained peak sEV fraction 5.5 ml (Figure 3(A,B)). Surprisingly, sEV-poor/protein-poor/triglyceride-rich fractions 4.5 ml and 5.0 ml demonstrated a similar degree of dye transfer to the peak sEV fraction (Figure 3(A,B)). Moreover, much higher (~10-fold) fluorescence was seen after incubation with sEV-poor/protein-rich fraction 8.0 ml (Figure 3(A,B)). Negligible dye transfer was obtained with a negative control in which PBS was used as a starting material (Figure 3(A)), demonstrating that free dye was effectively removed by the washing steps.

As the first indication that lipoproteins may contribute to dye transfer, overnight fasting of the animals led to lower lipophilic dye transfer with the early 4.5 ml and 5.0 ml fractions (Figure 3(A,B)).

### Masking of sEV uptake by contaminants is independent of sample source or lipophilic dye used

**Figure 4.**
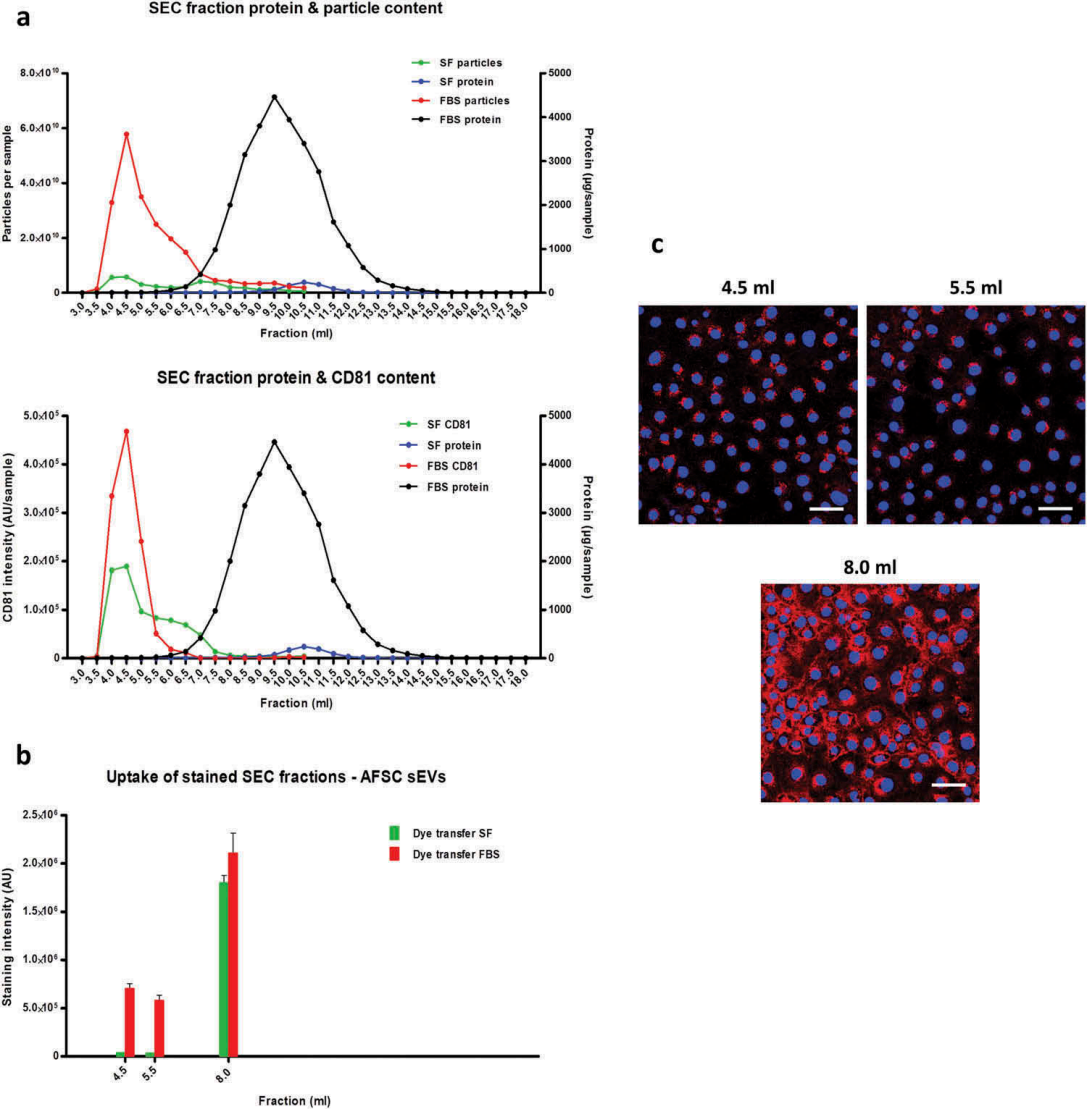
Uptake of lipophilic dye-labelled conditioned medium size-exclusion chromatography (SEC) fractions into mouse cardiac endothelial cells (MCECs). (A) Protein and particle amount (upper panel) and protein and CD81 amount (lower panel) of 0.5 ml SEC fractions collected from serum-supplemented or serum-free amniotic fluid stem cell (AFSC)-conditioned medium. Conditioned medium was precleared of cells, debris and larger vesicles and concentrated to < 500 µl before loading on a qEV SEC column; 0.5 ml fractions were collected and protein content was measured. Nanoparticle tracking analysis was performed on fractions up to 10.5 ml using an LM10-HS NanoSight instrument. CD81 protein marker was measured using a modified dissociation-enhanced lanthanide fluorescence immunoassay in SEC fractions 3.0–10.5 ml [22]. AU, arbitrary units measured by time-resolved fluorescence. Assays were performed together and AUs from serum-free and serum-supplemented samples can be compared. (B) Equal volumes (50 µl) of the indicated SEC fractions were labelled with 7.5 µg/ml CellMask Orange lipophilic membrane dye and washed. Small aliquots of the stained material (~15%) were added to a confluent layer of MCECs and incubated for 3 h. The graphs show means with SEM of fluorescence intensity of five images obtained in a single experiment. SF, serum-free conditioned medium; FBS, serum-supplemented conditioned medium (exosome-depleted foetal bovine serum). (C) Representative images of fluorescence uptake of SEC fractions (indicated) from serum-supplemented conditioned medium. Scale: 50 µm.

To ensure that these observations were not due to a characteristic specific to plasma, we performed similar experiments using conditioned cell culture medium using either serum-free or serum-supplemented medium (in this example we used AFSCs to isolate sEVs). SEC columns were able to clearly separate particles (quantified by NTA) from protein in conditioned medium samples, and these particles were confirmed as sEVs by the presence of CD81 marker (Figure 4(A)). sEV-rich fractions had higher CD81, particles and protein content in serum-supplemented samples compared to serum-free samples (Figure 4(A); Supplementary Figure 3(A)), possibly owing to the greater number of cells under serum-supplemented conditions (not shown). Similarly to plasma samples, vesicle-rich fraction 4.5 ml showed some dye transfer to recipient MCECs (Figure 4(A–C)). However, much greater cellular fluorescence uptake was observed with sEV-poor/protein-rich fraction 8.0 ml (Figure 4(A–C)). Furthermore, fraction 5.5 ml demonstrated far greater dye transfer when obtained from serum-supplemented than from serum-free conditions (Figure 4(B)), even though it contained a similar CD81 signal (Figure 4(A), lower panel).

Greater dye transfer with protein-rich fraction 8.0 ml compared to sEV-rich fraction 4.5 ml was also obtained with the commonly used PKH67 and DiD dyes, suggesting that these observations were independent of the type of lipophilic dye (Supplementary Figure 3(B,C)).

Overall, among all fractions, there was no correlation between sEV content and fluorescent dye uptake, indicating that any existing vesicular internalization may be masked by the presence of sample contaminants. This was further examined in subsequent experiments.

### Contaminants contribute to the uptake of lipophilic dye-labelled SEC fractions in primary cardiomyocytes

**Figure 5.**
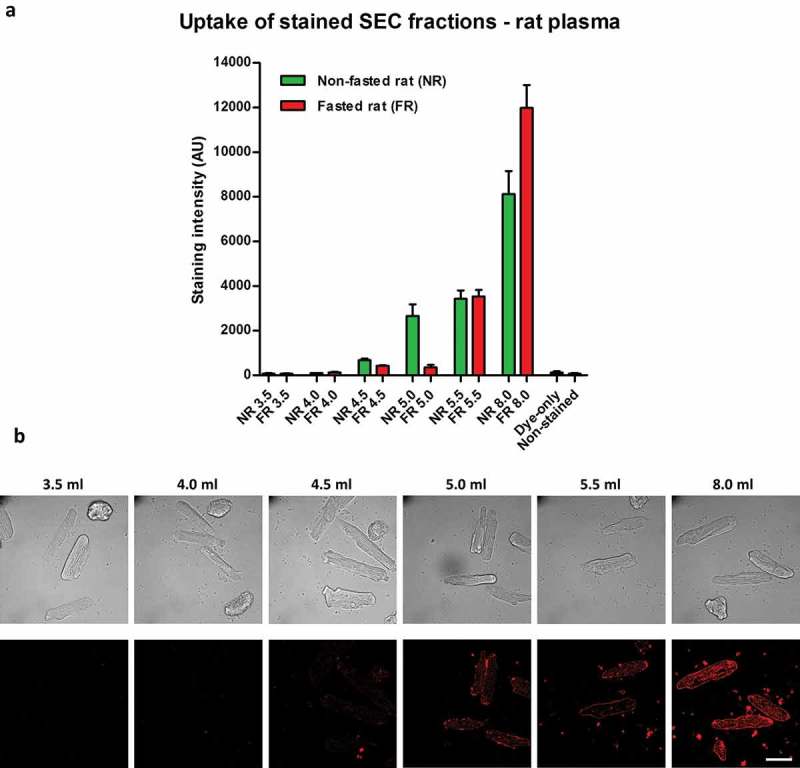
Uptake of lipophilic dye-labelled size-exclusion chromatography (SEC) fraction aliquots in primary adult rat cardiomyocytes. (A) Equal volumes (50 µl) of SEC fractions 3.5–5.5 ml and 8.0 ml obtained from fasted and non-fasted rat blood were labelled with 7.5 µg/ml CellMask Orange lipophilic membrane dye and washed. Small aliquots of the stained material (~15%) were added to primary rat cardiomyocytes and incubated for 3 h. The graphs show means with SEM of fluorescence intensity of five images obtained in a single experiment. NR, non-fasted rat; FR, fasted rat; followed by the respective SEC fraction in millilitres; Dye-only, phosphate-buffered saline negative control stained with the lipophilic dye; Non-stained, background cardiomyocyte fluorescence without addition of a sample; AU, arbitrary fluorescence intensity units. (B) Representative images of fluorescence uptake of SEC fractions from plasma collected from a non-fasted rat. Scale: 50 µm.

To further assess dye uptake, we used labelled rat plasma SEC fractions for the incubation of primary adult rat cardiomyocytes, which have been suggested to have no or minimal internalization of sEVs [31,32]. The pattern of uptake observed was similar to that in endothelial cells, with some dye transfer using sEV-rich fraction 5.5 ml but much greater fluorescent uptake with the sEV-poor/protein-rich fraction 8.0 ml (Figure 5(A,B)). In line with the endothelial cell data, fasting reduced staining with early fractions, which was most evident with fraction 5.0 ml (Figure 5(A)).

These results further support the findings that contaminants may mask the transfer of lipophilic dye-labelled sEVs.

### Fluorescence uptake of lipophilic dye-labelled sera in endothelial cells and primary cardiomyocytes

**Figure 6.**
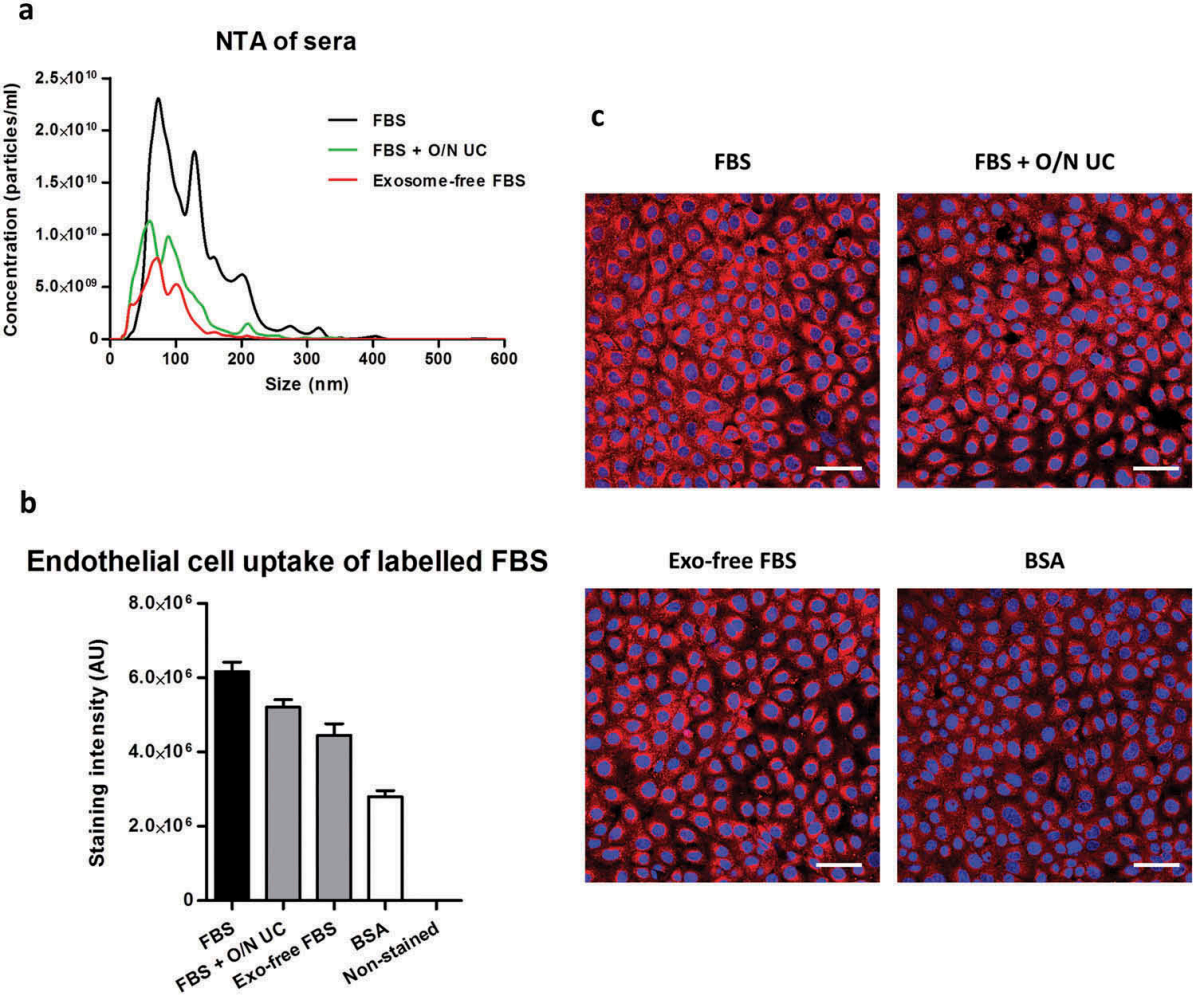
Uptake of lipophilic dye-labelled serum and protein-only samples in mouse cardiac endothelial cells (MCECs). (A) Particle size distribution of complete foetal bovine serum (FBS), in-house prepared exosome-depleted FBS by overnight ultracentrifugation (FBS + O/N UC) and commercially available exosome-depleted FBS (Exo-free FBS) measured by nanoparticle tracking analysis on an LM10-HS NanoSight instrument. (B) Equal volumes (20 µl) of each serum or a pure protein sample [bovine serum albumin (BSA); protein concentration equal to the complete FBS] were labelled with 7.5 µg/ml CellMask Orange lipophilic membrane dye and washed. Small aliquots of the stained material (~15%) were added to MCECs and incubated for 3 h. The graphs show means with SEM of fluorescence intensity of five images obtained in a single experiment. AU, arbitrary fluorescence intensity units; Non-stained, background MCEC fluorescence without addition of a sample. (C) Representative images of fluorescence uptake with sera and BSA samples. Scale: 50 µm.

To further assess the potential for dye to be transferred by components other than EVs, we used complete or exosome-depleted FBS for lipophilic dye labelling and studies of cellular uptake. When measured by NTA, both commercial and prepared in house, sEV-depleted sera contained greatly reduced particle concentrations compared to the complete FBS (Figure 6(A); Supplementary Figure 4(A)) as well as a markedly reduced protein concentration (Supplementary Figure 4(B)). Triglyceride content was similar in all the sera used (Supplementary Figure 4(C)).

To study the effect of proteins and lipoproteins on dye uptake, equal volumes of complete FBS, commercial exosome-depleted FBS or in-house-prepared exosome-depleted FBS were labelled with CellMask and added to MCECs. Compared to the complete FBS, there was a reduction of approximately 15% in stain transfer with the in-house-prepared exosome-depleted FBS and a reduction of approximately 28% with the commercially available exosome-depleted FBS (Figure 6(B,C)). Since the sEV-depleted sera also had a lower protein concentration (Supplementary Figure 4(B)), we directly investigated the protein contribution using a protein-only sample containing a concentration of BSA equivalent to the protein content of complete FBS (~33 mg/ml). Strikingly, the stained BSA was retained in the concentrate after 100 kDa filtration and its fluorescent stain transfer to endothelial cells accounted for around 46% of the complete FBS stain transfer (Figure 6(B,C)). A similar pattern was observed with uptake of labelled sera and protein-only samples in primary cardiomyocytes (Supplementary Figure 4(D,E)).

**Figure 7.**
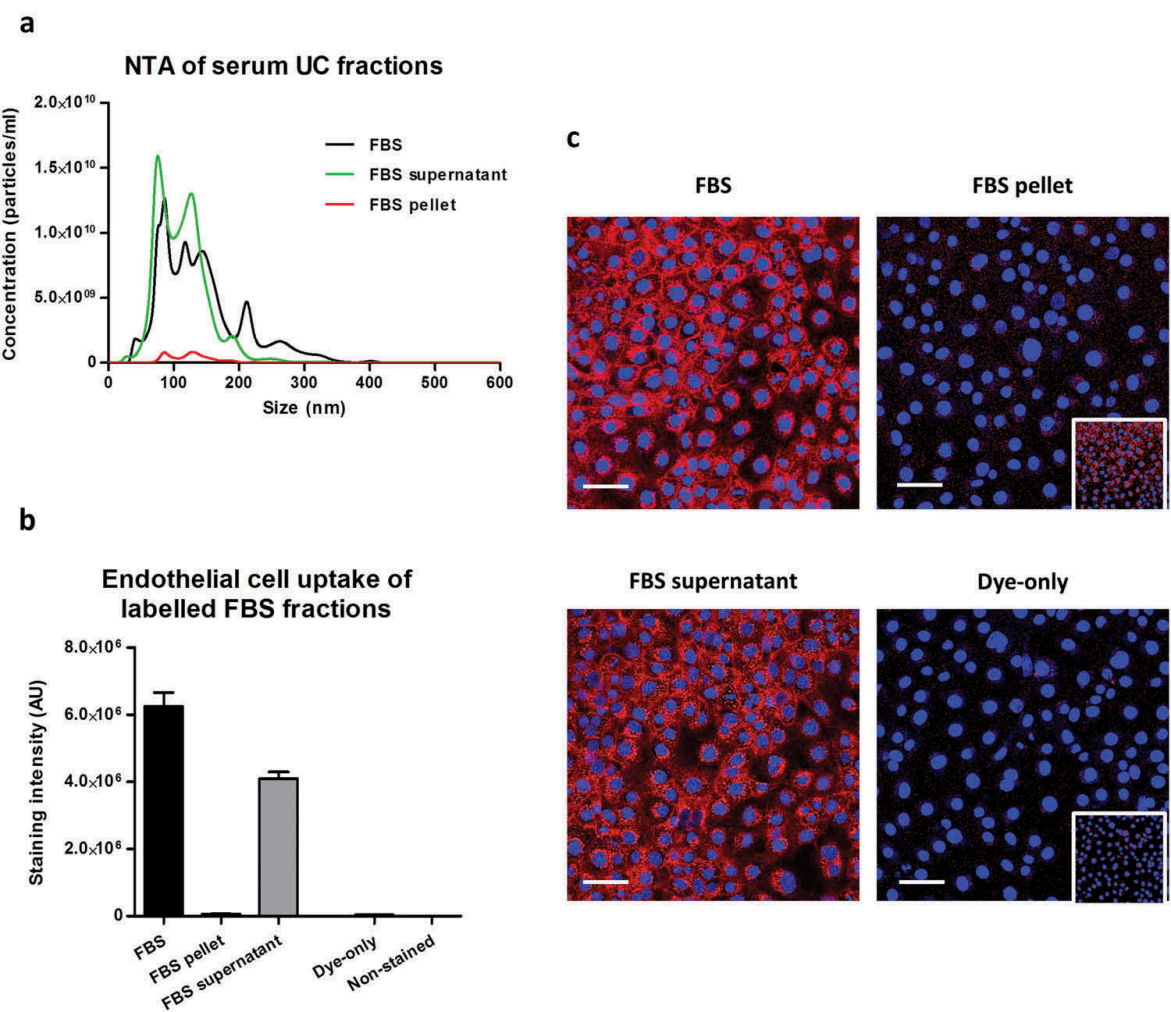
Uptake of lipophilic dye-labelled serum fractions in mouse cardiac endothelial cells (MCECs). (A) Particle size distribution of complete foetal bovine serum (FBS) as well as FBS supernatant and FBS pellet obtained after ultracentrifugation measured by nanoparticle tracking analysis on an LM10-HS NanoSight instrument. (B) Equal starting amounts of each fraction were labelled with 7.5 µg/ml CellMask Orange lipophilic membrane dye and washed. Small aliquots of the stained material (~15%) were added to MCECs and incubated for 3 h. The graphs show means with SEM of fluorescence intensity of five images obtained in a single experiment. AU, arbitrary fluorescence intensity units; Dye-only, phosphate-buffered saline negative control stained with the lipophilic dye; Non-stained, background MCEC fluorescence without addition of a sample. (C) Representative images of fluorescence uptake with serum fractions and Dye-only samples. Scale: 50 µm. Inserts at the bottom of FBS pellet and Dye-only samples are with equally increased contrast and intend to show the higher fluorescence with the FBS pellet.

Ultracentrifugation of complete commercial FBS was also used to separate it into an EV-rich FBS pellet and EV-poor/protein-rich/lipoprotein-rich FBS supernatant. FBS supernatant contained the majority of particles, measured by NTA, and the bulk protein of the complete FBS (Figure 7(A); Supplementary Figure 4(F,G)). CellMask staining showed that the amount of dye transfer after labelling the EV-rich FBS pellet was only about 1% of that when using complete FBS (Figure 7(B,C)). Conversely, dye transfer using the EV-poor FBS supernatant contributed to the majority of the complete FBS dye transfer (Figure 7(B,C)).

Taken together, these data suggest that vesicles may make only a minor contribution to fluorescent dye transfer to recipient cells compared to the contaminants present in a typical sEV isolate.

## Discussion

In this study, we used SEC to fractionate rat blood plasma or conditioned cell culture medium, and by means of lipophilic dye labelling we demonstrated that it is virtually impossible to discriminate between specific sEV uptake into an acceptor cell and contaminant artefacts. We argue that free protein and lipoproteins in the samples may make a significant contribution to the fluorescence transfer. Lipophilic dyes are not specific to sEVs and false-positive results may be observed with any isolation technique.

We observed the co-isolation of large amounts of APOB^+^ lipoproteins when using SEC to isolate sEVs from plasma. APOB is carried in most lipoproteins including chylomicrons, VLDLs, IDLs and LDLs but not high-density lipoprotein (HDL) [51]. Our findings support previous reports showing that SEC [22] and ultracentrifugation [21] of plasma lead to co-isolation of vesicles and APOB^+^ lipoproteins. It was demonstrated that LDL particles and sEVs or microvesicles may be physically associated [21]. Although methods such as density gradient centrifugation may provide better separation of sEVs from most lipoproteins, this method is still not able to separate sEVs from HDLs owing to their similar densities [17]. Thus, complete separation of lipoproteins and vesicles from blood or conditioned tissue culture medium containing serum is unlikely to be achieved, regardless of the method used.

We observed lower particle, triglyceride and APOB levels in fractions 4.5 ml and 5.0 ml of SEC-processed plasma collected from a fasted rat, which could be due to a reduced number of large lipoproteins. These reductions corresponded to a lower fluorescence transfer to recipient cells compared to a non-fasted rat. Taking this into account, along with the relatively low protein levels and sEV markers, suggests that lipoproteins may be the main contributors to dye transfer in early SEC fractions. However, such an effect was not evident with sEV-rich fraction 5.5 ml, which demonstrated similar dye transfer independent of fasting. A possible explanation for this is the higher protein, APOB and tetraspanin markers in this fraction, indicative of a more complex mixture of free protein, lipoproteins and vesicles. Moreover, the use of various sEV-poor fractions, serum and free protein samples clearly showed that the lipid dyes can bind to components other than EVs. Therefore, it may be difficult to discriminate between the relative contribution of free protein, lipoproteins and vesicles to the fluorescent dye transfer of the sEV-rich fraction 5.5 ml.

Indeed, previous reports have used lipophilic dye labelling to investigate the cellular uptake of various lipoprotein particles including VLDL [44], LDL [40–42] and HDL [43,45]. Our experiments with stained FBS samples further support the observations that lipoproteins may be labelled by a lipophilic dye. Importantly, this has implications not only for blood-derived sEVs but also for sEVs isolated from conditioned cell culture media supplemented with serum, as seen with our experiments. A 75 cm^2^ tissue culture flask with 10% serum supplementation will contain 1–1.5 ml of serum, which may cause a marked lipoprotein contamination in downstream experiments with vesicles. Taken together, this suggests lipoproteins may produce false positives when using lipophilic dyes to label isolated vesicles.

A common approach to control for artefacts in internalization studies includes the use of a vehicle, such as PBS, processed similarly to the sample of interest to ensure that the observed recipient cell fluorescence uptake is not caused by carryover of unbound dye [33]. We used 100 kDa filters, which efficiently removed the excess dye from a PBS sample as shown by the negligible transfer of fluorescence to cells. To our surprise, dye retention by a protein-only BSA sample was significant after 100 kDa filtration even when a few additional washes with PBS were performed. BSA has a molecular weight of approximately 66 kDa [52] and the majority is expected to be removed after 100 kDa filtration, but our results indicate aggregate formation and/or inability of the filter to remove BSA–dye complexes. It has been argued that albumin can interact directly with vesicles since a 120 ml large-scale SEC column or ultracentrifugation was unable to completely separate albumin from vesicles of plasma [47]. We further showed that plasma sEV-poor/protein-rich fraction 8.0 ml can transfer around 10 times more fluorescence to recipient cells than the sEV-rich 5.5 ml fraction. A similar pattern was evident using primary isolated cardiomyocytes.

While the elution profile of sEV from conditioned medium matched very closely with that illustrated in the manufacturer’s instructions (Izon, qEV: http://www.izon.com/exosome-isolation/), the elution profile of particles from blood plasma was comparably delayed. This discrepancy could be attributed to the use of rat plasma in our study versus serum in the published manufacturer’s instructions, which is devoid of a large amount of coagulation proteins. In addition, rat plasma is more viscous than human plasma [53] and viscosity is crucial for the resolution of SEC [54]. Furthermore, the manufacturer’s guidance reports tunable resistive pulse sensing results for particle counting, whereas we assessed the samples by NTA. This factor could explain the presence of a large number of particles detected in later fractions from SEC of plasma, as reported by others [22].

Our experiments with conditioned medium confirmed the results obtained with plasma showing the highest dye transfer with sEV-poor/protein-rich SEC fraction 8.0 ml. Crucially, this was also observed with serum-free conditioned medium, suggesting that protein–dye binding is not restricted to high-binding plasma proteins (e.g. albumin). It should be noted that the markedly higher protein in fraction 8.0 ml of the serum-supplemented sample compared to the serum-free sample corresponded to only a slight increase in dye transfer. This is most likely to be due to the very intense staining and a saturation of the dye uptake. Another possibility is that a component of the conditioned medium eluting in late fractions (e.g. fraction 8.0 ml) causes an artefact dye transfer independent of the serum supplementation. There are indications that vesicles may contribute little to dye transfer since conditioned medium fraction 5.5 ml demonstrated much more staining in the serum-supplemented sample than in the serum-free sample, despite containing a similar CD81 signal. Instead, greater dye transfer corresponded to a higher particle and protein content of this fraction, further supporting protein–dye and/or lipoprotein–dye binding. This effect could also be partly due to an inherent difference in vesicles, as their protein cargo may be altered by serum-free conditions [55] which could affect subsequent uptake. Nevertheless, further support for contaminant artefacts is the mismatch between serum-supplemented fractions 4.5 ml and 5.5 ml, where a similar degree of dye transfer was observed regardless of the greater than nine times higher CD81 signal for fraction 4.5 ml.

In our experiments with labelled serum samples, we observed that sEV depletion may reduce the fluorescence transfer of stained sera, but this effect could be attributed to the substantial removal of protein content (45–67%) rather than the sEV depletion itself. The non-specific nature of the lipophilic dyes was further shown by the experiments with staining of FBS fractions after ultracentrifugation. We demonstrated that the FBS ultracentrifugation pellet contributed to only a fraction of the dye transfer of the complete FBS, the majority of this staining coming from components of the FBS ultracentrifugation supernatant.

In this study we did not observe any particular differences in the pattern of intracellular versus membrane fluorescence between the uptake of sEV-rich and sEV-poor samples. Fluorescence was mostly intracellular and close to the nucleus in MCECs, while in cardiomyocytes it was seemingly confined to the cell membrane. This is potentially due to the nature of these primary cells, which have lower propensity for membrane recycling.

The PKH67 dye manufacturer’s instructions explicitly recommend using BSA as a dye-scavenging agent [46]. Given the inability to clear BSA–dye complexes with 100 kDa filters and the likelihood of pelleting some BSA with vesicles after ultracentrifugation, it seems highly probable that protein–dye carryover will make a significant contribution to fluorescence transfer in lipophilic dye-labelling experiments. Our findings seem not to be limited by a characteristic non-specificity of CellMask dye since we obtained similar results with PKH67 and DiD dyes, which bound the free proteins and/or lipoproteins in fraction 8.0 ml from serum-supplemented conditioned medium and transferred the dye to the recipient cells.

Other authors also report false positives with lipophilic dye labelling of vesicles. For instance, Lai et al. observed approximately 2.5 times higher *in vitro* fluorescence transfer of PKH67-stained, vesicle-depleted conditioned medium than PKH67-stained isolated vesicles [39]. As pointed out by the authors, PKH67 may label non-specifically other medium components. They also used genetic labelling of vesicles using cell transfection with a membrane-targeted fluorescent protein [39]. They demonstrated that this method produces a much lower signal than PKH67-labelled vesicles, implying the non-specificity of PKH67 staining.

There are some examples where an inhibition of uptake was observed after vesicle labelling with lipophilic dyes. For instance, low temperature (4°C) may inhibit internalization of vesicles stained by a lipophilic dye [36,56]. It should be noted, however, that endocytosis and membrane recycling occur poorly, if at all, at temperatures below 10°C [57,58], suggesting that this effect may not be specific to sEVs. Moreover, the use of pharmacological inhibitors of known import pathways may not be specific to the uptake of vesicles (reviewed in [25]). For example, dynasore, an inhibitor of clathrin-dependent endocytosis [59], can also block LDL cellular uptake [60]. Hence, inhibition of uptake cannot discriminate between internalization of labelled vesicles, lipoproteins or protein–dye binding and carryover.

Other methods for sEV labelling exist which may provide a more specific way of tracking the vesicles’ fate. For example, cell transfection with a fusion construct of a fluorescent protein and an sEV-specific marker (e.g. CD63-GFP [61,62]) or transfection with a membrane-targeted fluorescent or reporter protein are such possibilities [39,63]. Unfortunately, genetic labelling cannot be performed with plasma sEVs such as isolates from human blood. Recently a Cre-*loxP*-based method was also used as a reliable way for assessment of *in vitro* or *in vivo* uptake of EVs [64]. This approach used transfection of a donor cell with Cre recombinase, which is incorporated in vesicles and subsequently tracked by *loxP* excision-dependent reporter expression in recipient cells [64].

A limitation of our study is that we have not determined with certainty the precise relative proportion of lipophilic dye binding to different types of lipoprotein particles, protein contaminants or the vesicles in our samples. Nevertheless, the use of both plasma and conditioned medium and three different lipophilic dyes provides solid evidence for the lack of specificity of this method. Importantly, we used sEV-rich and sEV-poor but protein- and/or lipoprotein-rich SEC fractions from the same isolation, providing internal controls normally not performed in uptake studies which only use single sEV-rich samples.

In conclusion, we demonstrate that lipophilic dyes may not be reliable sEV labelling agents unless an entirely pure population of sEVs is obtained that is completely devoid of protein and lipoprotein particles. Conclusions drawn from these experiments have to be interpreted with caution and multiple and appropriate controls should always be included (e.g. free protein sample with a concentration equal to sEV sample protein concentration). Ideally, alternative approaches should be sought for specific vesicle labelling such as cellular transfection with vesicle-targeted fluorescent or reporter proteins.
