# Supplementary material for: Confounding factors in vesicle uptake studies using fluorescent lipophilic membrane dyes
Source: J Extracell Vesicles. 2017 Oct 12;6(1):1388731. doi: 10.1080/20013078.2017.1388731 (PMC5699187; doi:10.1080/20013078.2017.1388731)
Supplement: Supplementary_Figures.zip [file ZJEV_A_1388731_SM2966.zip › Supplementary Fig Legs.docx]

**Supplementary Figure 1**

Particle size distribution in early fractions obtained with SEC of rat blood plasma.

NTA was performed on an LM10-HS NanoSight instrument. Particle size distribution is shown for SEC fractions 4.5 ml, 5.0 ml and 5.5 ml obtained from fasted or non-fasted rat blood plasma.

**Supplementary Figure 2**

Electron microscopy of early and late fractions obtained with SEC of rat blood plasma.

(A) TEM of early 4.5 ml SEC fraction obtained from non-fasted (left) and fasted (right) rat blood plasma. Scale: 1 µm. (B) TEM of late 8.0 ml SEC fraction obtained from non-fasted plasma. Scale: 100 nm.

**Supplementary Figure 3**

Protein content of early conditioned medium-derived SEC fractions and uptake of PKH67- or DID-labelled fractions in MCECs.

(A) Detail of protein content in early fractions (3.0-5.5 ml) from Figure 4A. Conditioned medium was pre-cleared of cells, debris and larger vesicles and concentrated to < 500 µl before loading on a qEV SEC column. 0.5 ml fractions were collected and protein content measured. (B) Representative images of PKH67 stained SEC fractions (indicated) uptake into MCECs. Equal volumes (100 µl) of each indicated SEC fraction were labelled with 3 µM PKH67 dye. Small aliquots of the stained material (~15%) were added to a confluent layer of MCECs and incubated for 3 hours. Right panels are zoomed in sections of the left panels. Scale: 50 µm. (C) Representative images of DiD stained SEC fractions (indicated) uptake into MCECs. Equal volumes (100 µl) of each indicated SEC fraction were labelled with 5 µM DiD dye. Small aliquots of the stained material (~15%) were added to a confluent layer of MCECs and incubated for 3 hours. Right panels are zoomed in sections of the left panels. Scales: 50 µm (left panels); 20 µm (right panels).

**Supplementary Figure 4**

Particle, protein and triglyceride concentration of FBS and uptake of lipophilic dye-labelled sera and protein samples in primary rat cardiomyocytes.

(A) Particle concentration in complete FBS (“FBS”), in-house prepared exosome-depleted FBS by overnight ultracentrifugation (“FBS + O/N UC”) and commercially available exosome-depleted FBS (“Exo-free FBS”) measured by NTA on an LM10-HS NanoSight instrument. (B) Sera protein concentrations measured by a BCA assay. (C) Sera triglyceride concentrations measured with a commercial kit. (D) Equal volumes (20 µl) of each serum or a pure protein sample (bovine serum albumin, BSA; protein concentration equal to the complete FBS) were labelled with 7.5 µg/ml CellMask Orange lipophilic membrane dye, washed and small aliquots of the stained material (~15%) were added to primary rat cardiomyocytes. Stain transfer was measured after 3 h of incubation. AU – arbitrary fluorescence intensity units. The graphs show means with SEM of fluorescence intensity of five images obtained in a single experiment. (E) Representative image of cardiomyocyte uptake of labelled complete FBS showing staining of the sarcolemma. Scale: 20 µm. (F) Particle amount used for staining in complete FBS as well as FBS pellet and FBS supernatant after ultracentrifugation measured by NTA on an LM10-HS NanoSight instrument. (G) Protein amount used for staining in complete FBS as well as FBS pellet and FBS supernatant after ultracentrifugation measured by a BCA assay.
