# Supplementary figures and images for: Confounding factors in vesicle uptake studies using fluorescent lipophilic membrane dyes
Source: J Extracell Vesicles. 2017 Oct 12;6(1):1388731. doi: 10.1080/20013078.2017.1388731 (PMC5699187; doi:10.1080/20013078.2017.1388731)

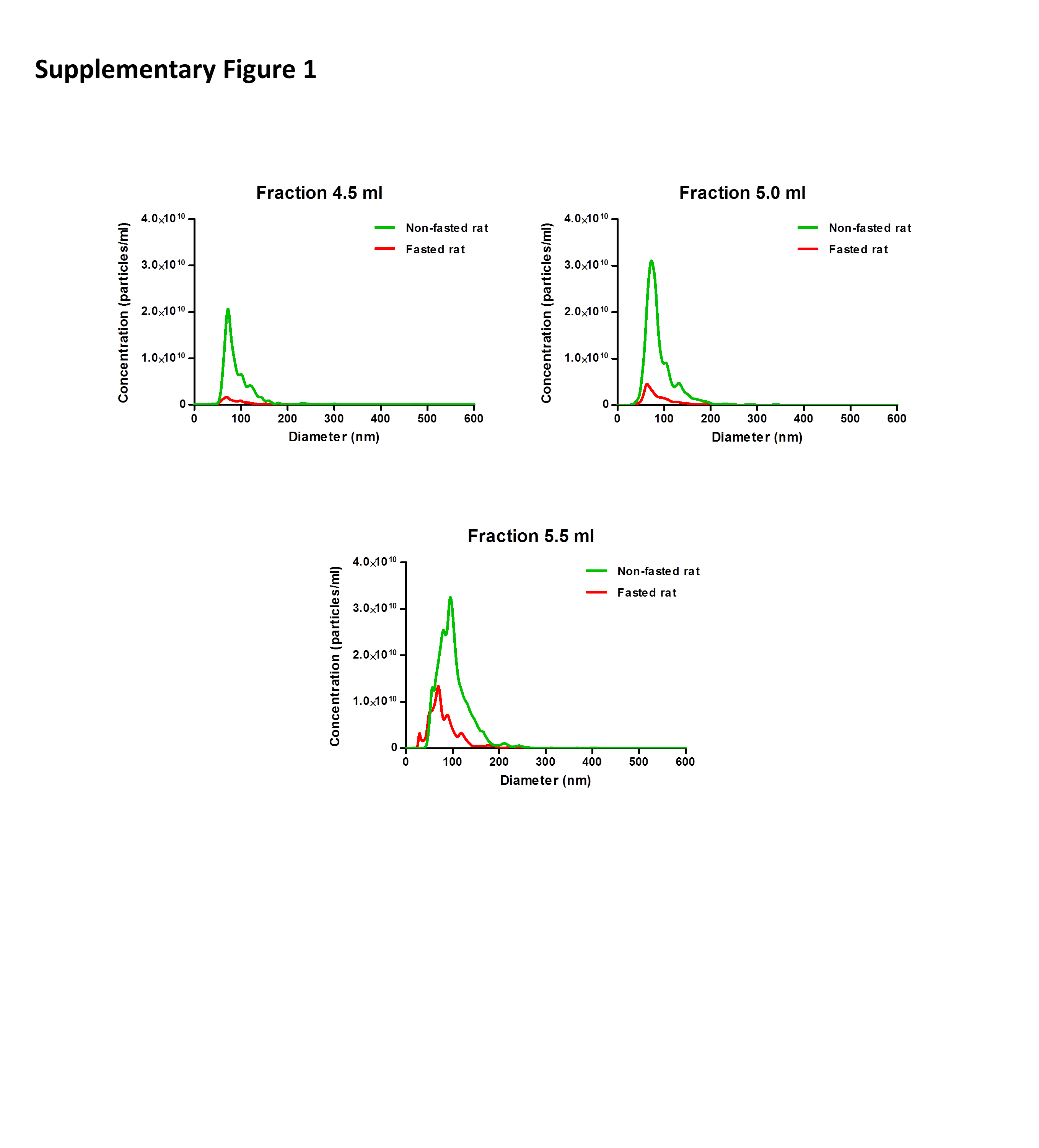

Supplement: Supplementary_Figures.zip [file ZJEV_A_1388731_SM2966.zip › Supplementary Figure 1.TIF]

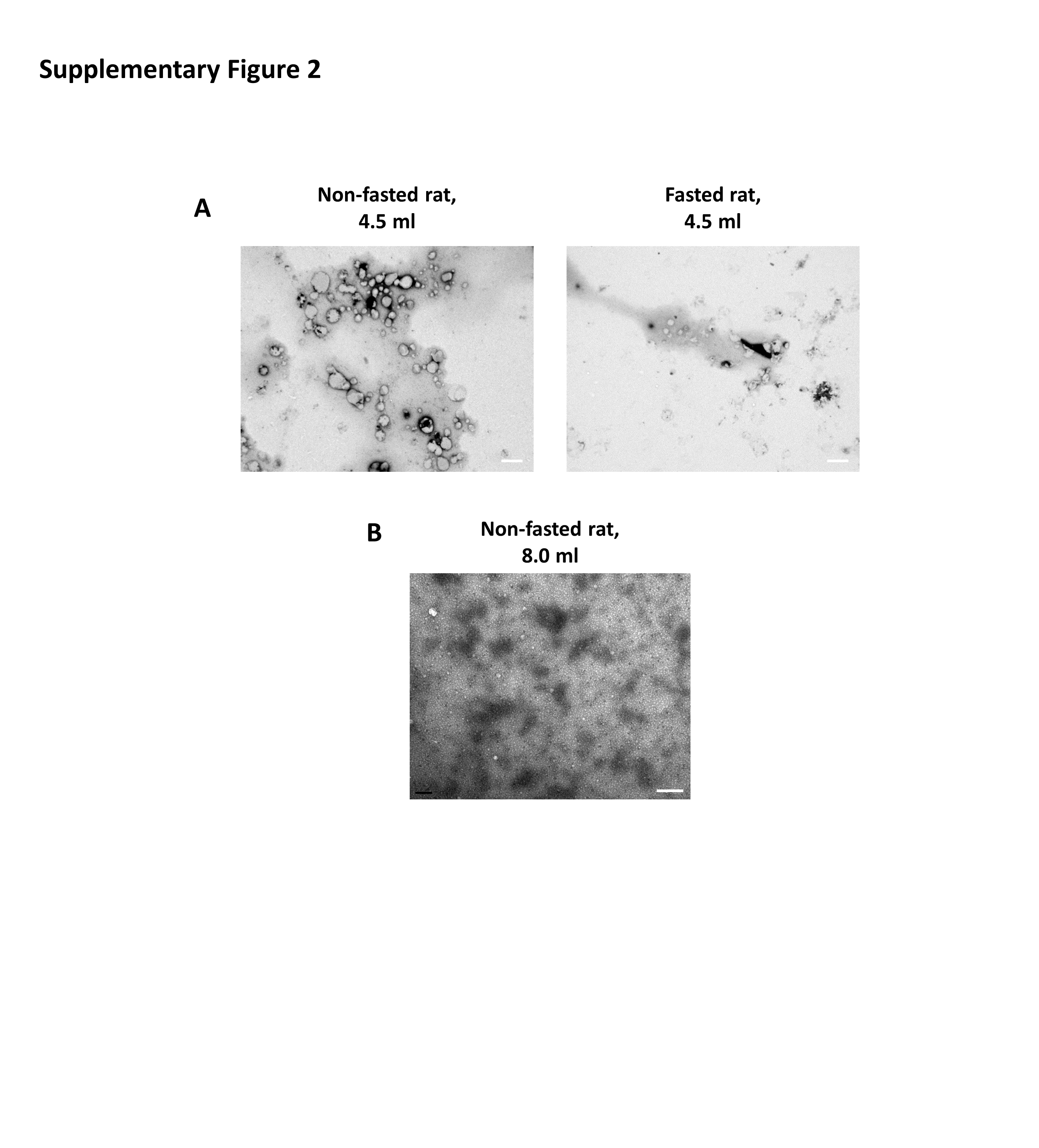

Supplement: Supplementary_Figures.zip [file ZJEV_A_1388731_SM2966.zip › Supplementary Figure 2.TIF]

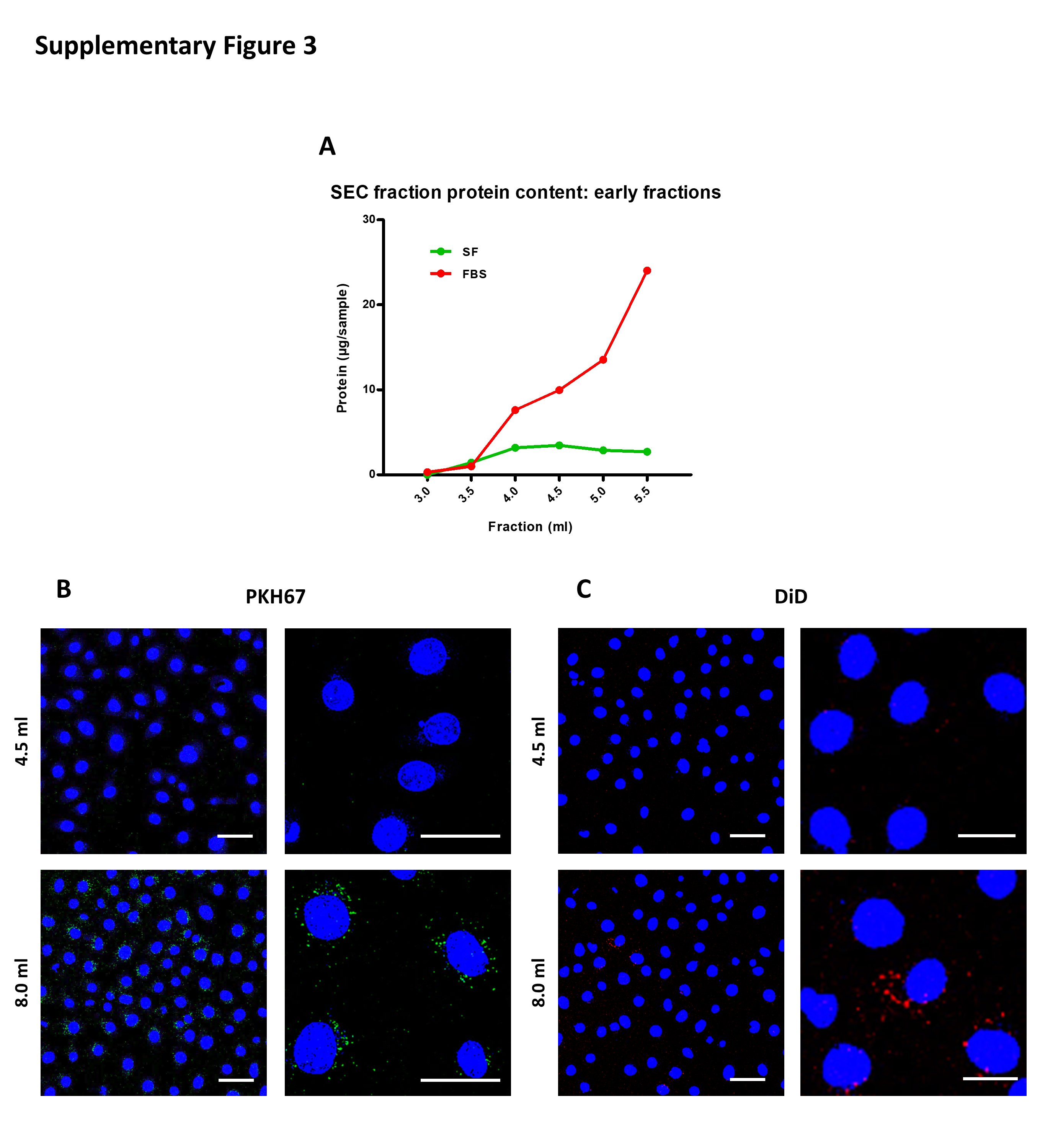

Supplement: Supplementary_Figures.zip [file ZJEV_A_1388731_SM2966.zip › Supplementary Figure 3.tif]

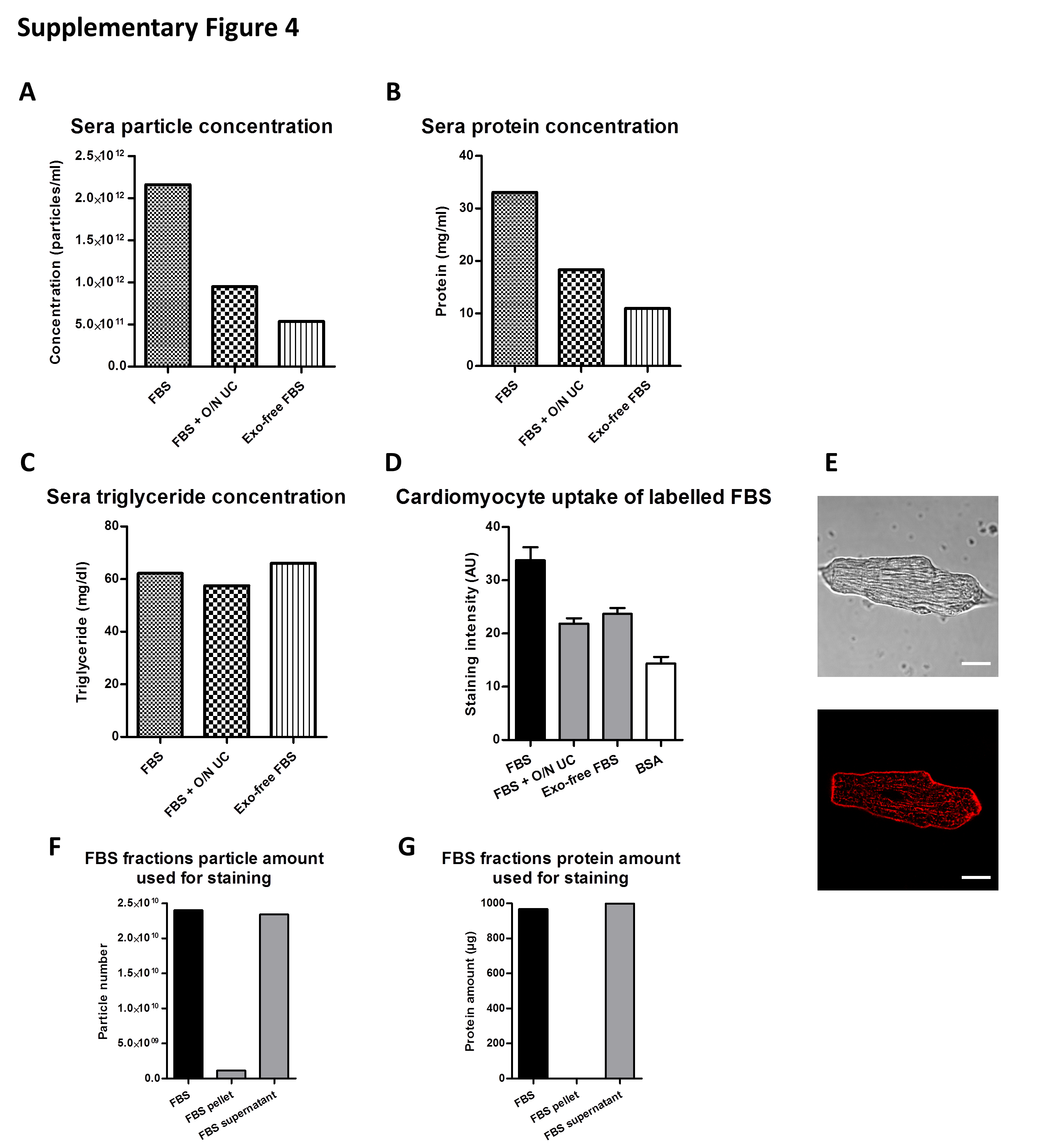

Supplement: Supplementary_Figures.zip [file ZJEV_A_1388731_SM2966.zip › Supplementary Figure 4.TIF]
